# The ABRF Metabolomics Research Group 2016 Exploratory Study: Investigation of Data Analysis Methods for Untargeted Metabolomics

**DOI:** 10.3390/metabo10040128

**Published:** 2020-03-27

**Authors:** Christoph W. Turck, Tytus D Mak, Maryam Goudarzi, Reza M Salek, Amrita K Cheema

**Affiliations:** 1 Max Planck Institute of Psychiatry, Kraepelinstr. 2, 80804 Munich, Germany; turck@psych.mpg.de; 2 Mass Spectrometry Data Center, National Institute of Standards and Technology, 100 Bureau Drive, Gaithersburg, MD 20899, USA; tytus.mak@nist.gov; 3 Cardiovascular and Metabolic Sciences, Lerner Research Institute, The Cleveland Clinic Foundation, 9500 Euclid Ave, Cleveland, OH 44195, USA; goudarm@ccf.org; 4 International Agency for Research on Cancer, 150 court Albert Thomas, 69372 Lyon CEDEX 08, France; SalekR@iarc.fr; 5 Department of Oncology, Lombardi Comprehensive Cancer Center, Georgetown University Medical Center, Washington, DC 20057, USA; 6 Departments of Oncology and Biochemistry, Molecular and Cellular Biology, Georgetown University Medical Center, Washington, DC 20057, USA

**Keywords:** metabolomics, “omics” analyses, data preprocessing

## Abstract

Lack of standardized applications of bioinformatics and statistical approaches for pre- and postprocessing of global metabolomic profiling data sets collected using high-resolution mass spectrometry platforms remains an inadequately addressed issue in the field. Several publications now recognize that data analysis outcome variability is caused by different data treatment approaches. Yet, there is a lack of interlaboratory reproducibility studies that have looked at the contribution of data analysis techniques toward variability/overlap of results. The goal of our study was to identify the contribution of data pre- and postprocessing methods on metabolomics analysis results. We performed urinary metabolomics from samples obtained from mice exposed to 5 Gray of external beam gamma rays and those exposed to sham irradiation (control group). The data files were made available to study participants for comparative analysis using commonly used bioinformatics and/or biostatistics approaches in their laboratories. The participants were asked to report back the top 50 metabolites/features contributing significantly to the group differences. Herein we describe the outcome of this study which suggests that data preprocessing is critical in defining the outcome of untargeted metabolomic studies.

## 1. Untargeted Metabolomics: An Emerging Technology for Biomarker Discovery

Liquid chromatography–mass spectrometry (LC-MS)-based untargeted metabolomics and lipidomics technologies are used for delineating precise changes in metabolism that can be correlated with a phenotype. Untargeted metabolomics offers researchers an unprecedented chance to make new discoveries that provide novel insights into disease onset and progression, using a wide array of biological matrices. Additionally, the high throughput nature of LC-MS-based metabolomics allows for the analysis of large batches of samples. From a clinical standpoint, biofluid-based biomarkers have a high clinical translation potential after suitable validation studies.

Several publications now recognize that data analysis outcome variability is caused by different data treatment approaches [1,2,3]. Yet, there is a lack of interlaboratory reproducibility studies that have investigated the contribution of data analysis techniques toward variability/overlap of results.

## 2. Association of Biomolecular Research Facilities (ABRF) Metabolomics Research Group (MRG) 2016 Study Design

The Metabolomics Research Group (MRG) designed an interlaboratory data analysis study to recapitulate a typical metabolomics experiment where the participants would use any data analysis package of their choice to report back the top 50 most statistically significant perturbed spectral features between two study groups. In untargeted metabolomics, the raw MS data is routinely preprocessed using either vendor-based software (depending on the MS type) or using open source software (e.g., MZmine or XCMS). For the latter, the raw data files have to be converted to a universal reading format like mzxml or netCDF. The preprocessed data files (typically in csv format) are then used for statistical/bioinformatics analysis wherein normalized intensities for the spectral features are compared between and across study groups to select features that contribute significantly to group differences using a statistical test cutoff value (i.e., *p*-value < 0.05), followed by false discovery rate (FDR) correction. The shortlisted features are then subjected to accurate mass-based database searches for putative annotations. Several publicly available databases including Metlin [4], HMDB (Human Metabolome Database) [5], MMCD (Madison Metabolomics Consortium database) [6], and CEU Mass Mediator [7] enable an automated batch search for a given list of features. Once the features are putatively annotated, their identities need to be validated using MS/MS fragmentation and retention time confirmation and/or using pure reference standard compounds. While these general steps are traditionally followed by most laboratories, the software and data analysis criteria used vary among the laboratories.

**Figure 1:**
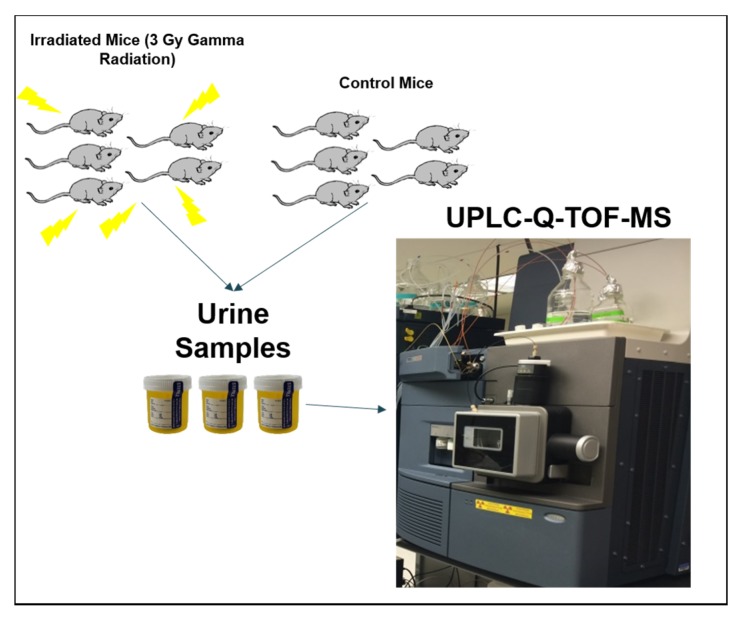
Study Design Schema.

To do this, we used an untargeted, comparative metabolomics approach using urine samples from C57BL6 mice exposed to 3 Gy of external beam gamma rays and those exposed to sham irradiation (control group) (Figure 1). We used five biological replicates for each group. A total of 11 urine samples including 5 controls, 5 irradiated samples, and 1 pooled urine sample (used as quality control; QC) were used in this study. The pooled QC was prepared by combining 5 µL of each sample extract from the two groups. The urine samples were processed for mass spectrometry analysis by extraction with a solvent containing 30% acetonitrile, 40% methanol, and 30% water and removal of precipitated proteins by centrifugation (Supplementary Figure S1). The urine extracts were resolved on an H-class Acquity Ultra-performance liquid chromatography (UPLC) using reversed-phase chromatography (see Supplementary Figure S2 for gradient details). Data were acquired on a quadrupole time-of-flight mass spectrometer operating in positive or negative ionization modes. The sample queue was randomized to remove injection bias. A mixture of standard compounds (referred to as metmix composed of 1 ug/mL acetominophen, val-tyr-val, sulfaguanidine, sulfadimethoxine, leucine-enkephalin, terfenadine) was injected at the beginning and end of the sequence to monitor mass accuracy. Mass accuracy was within 5 ppm during batch acquisition (Supplementary Figure S3). We also performed blank (solvent only) injections to monitor carryover (Supplementary Figure S4) and used pooled quality control samples to monitor data reproducibility and retention time drift (Supplementary Figure S5). The examination of QC sample TIC overlays, metmix mass accuracy, and blank runs allowed us to conclude that the metabolomics data were robust.

We invited metabolomics researchers by announcing the study on the MRG homepage (https://abrf.org/sites/default/files/2016_mrg_study_outline_060716.pdf), contacting core laboratory directors, and advertising the study at the ABRF and American Society of Mass Spectrometry annual meetings. We asked the participants to analyze the data, available for download on Bioshare (https://bioshare.bioinformatics.ucdavis.edu/bioshare/view/tthvputgo2am4yo/), employing methods that are routinely used in their laboratory. The MRG also collected fragmentation data using MSE (elevated collision energy) with the QC samples and these files were available upon request for metabolite identification based on fragmentation patterns. For this purpose, we made the raw data as well as netCDF files available to provide more flexibility for data preprocessing. However, we expected that only those participants who use Waters software (Mass Lynx v4.0, Waters Corporation, Milford, MA, USA) would be able to take advantage of raw data. We asked the participants to report on the top 50 spectral features that show statistically significant differences in their relative abundances in the irradiated group compared to the sham group. The overarching goal was to inform the scientific community about the current status of metabolomics data analysis strategies. Specifically, we wanted to find out the possible causes of variability in data preprocessing routines, data postprocessing software, parameters used, choice of data filtering and imputation, feature selection, and metabolite identification.

**Figure 2:**
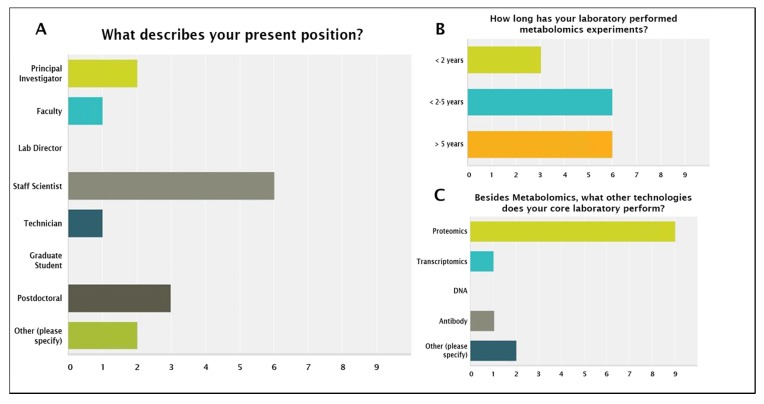
MRG study participants’ information.

**Figure 3:**
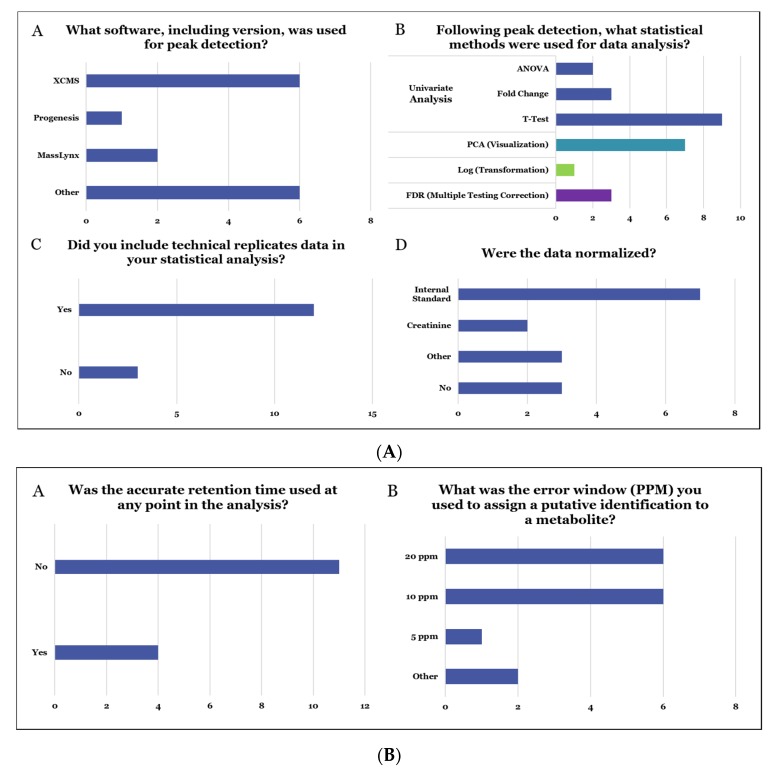
(**A**) Details on data analysis methodologies across participant laboratories for data processing (Panel A), statistical analysis (Panels B and C), and data normalization (Panel D). (**B**) LC-MS data parameters including RT (Panel A) and mass accuracy (Panel B) used by participants for accurate mass-based database search.

We also conducted an online survey to get information about participants’ laboratories and expertise as well as methodologies used for data analysis (https://www.surveymonkey.com/r/abrfmet2016). Additionally, MRG requested each participant to prepare a short write-up that summarized the approach that was taken, the methods that were used, and the key findings that were obtained (Supplementary Files 1–8). The write-ups provided each participant with the opportunity to communicate their results in their own words and share important details about the analysis that may not have been captured in the online survey. The geographical distribution of participants included four from the United States, three from Germany, and one from Switzerland from independent laboratories and/or institutions. A summary of the survey data is shown in Figure 2 and Figure 3. The majority of the participants were staff scientists, followed by postdoctoral fellows, faculty, and technicians with an average experience of >2.5 years in the metabolomics field. The majority of the participants also offered proteomics services, which seems to be a common trend in biomolecular core facilities at academic institutions (Figure 2, Panel C).

The MRG requested the following from the participants:

- Deconvolute the raw data using a preprocessing software of their choice and provide a data matrix consisting of *m*/*z*, retention time, and ion intensity (e.g., peak area);
- Postprocess the data using statistical tools of their choice and determine the top 50 most statistically significant perturbed (e.g., *p*-value) mouse urinary spectral features postexposure to 5 Gy external beam irradiation;
- Assign putative identification to these urinary spectral features using various online databases.

As shown in Figure 3, the majority of the participants either used XCMS or other software for peak picking (Panel A), different statistical tests for feature selection including PCA, student’s t-test, ANOVA, and so forth (Panel B), technical replicates in software-based data analysis (Panel C), and normalized data to the internal standard (Panel D). In addition, the majority of the participants did not use retention time, but stringent database search criteria for metabolite annotation (Figure 3B). The databases used included Human Metabolome Database (HMDB), KEGG, and CEU Mass Mediator. The results were submitted online or via email in an anonymous format. In addition to the deliverables above, we requested from those participants who used the raw data that they submit intermediate files containing data-processing parameters and small molecule identifications and/or quantitative values (i.e., abundance) for extracted peaks. This information enabled us to separately assess the impact of identification and/or peak detection from alternative sources in tandem with the integration of the results. Taken together, the MRG sought to get the following detailed information from the study:

- Methods used for the determination of relative quantitative metabolite differences across the two sample types (groups).
- The effects of different computational techniques on the determination of significantly altered metabolites in the two groups.
- The level of confidence and consistency in the results obtained from unique computational and chemometric approaches.
- The ability of software to determine differences across samples or help analyze data from metabolomics experiments.
- Databases used for assigning metabolite ID.

## 3. Meta-Analysis of Study Participant Results

**Figure 4:**
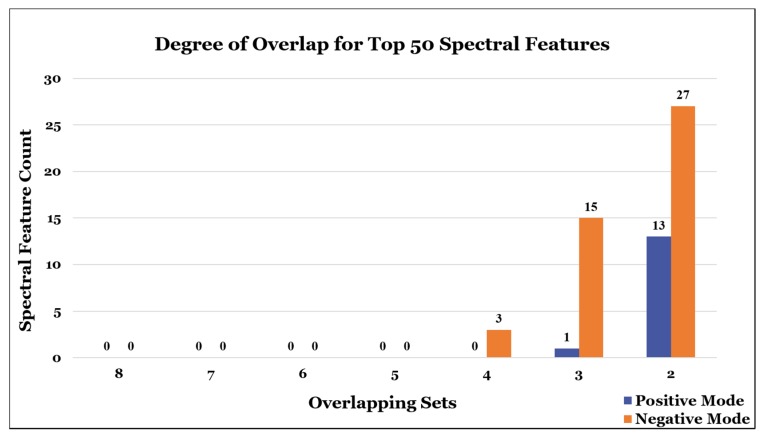
Overlap of the top 50 spectral features. The total number of overlapping features found between the lists of the top 50 most perturbed spectral features from each of the 8 participants, as a function of the number of groups they were found in. There were no features found to be shared by all 8 lists. When 4 lists were considered, 3 negative mode features were found to be shared. Overall there was far more overlap in the negative mode lists, with a total of 15 features shared by any combination of 3 lists, and 27 features by any combination of 2 lists. Only 1 feature was found to be shared by any 3 lists in the positive mode, and 13 features found to be shared by any combination of 2 lists.

The lists of the top 50 most perturbed spectral features submitted by each of the eight participants were analyzed for concurrence via *m*/*z* value and retention time comparisons. An agglomerative bottom-up approach was utilized for identifying similar spectral features. Every feature in each list was initially considered as its own cluster. Clusters were merged if any two constituent features matched to within 50 ppm of their *m*/*z* values and +/− 20% of their retention times. The results of this agglomerative clustering approach allowed for a simple but comprehensive interpretation of the study by means of tallying the number of overlapping spectral features found as a function of the number of participant list results they were found in. No spectral features were found to appear in all eight lists, and overlap was not observed until four lists were considered. While only three common negative mode spectral features were found in any combination of four out of the eight lists, none were found for positive mode features among any combination of five, six, or seven lists (Figure 4).

**Figure 5:**
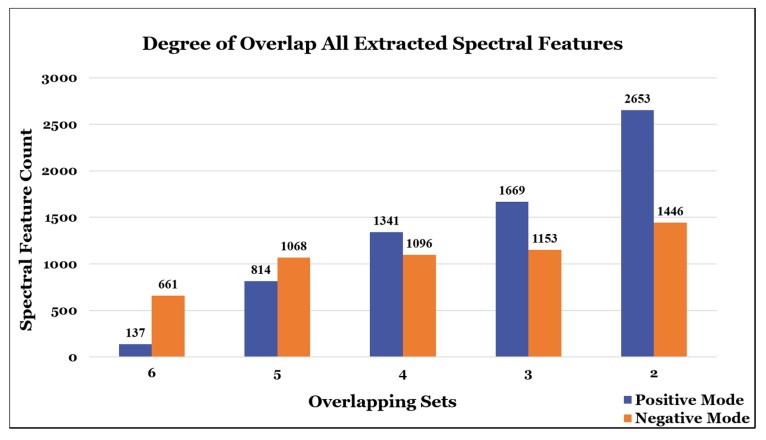
Degree of overlap for all extracted spectral features. The graph shows the total number of overlapping features found between the full extracted list of spectral features from 6 of the participants that provided raw data. More overlap was observed in the negative mode data compared to positive mode data.

**Figure 6:**
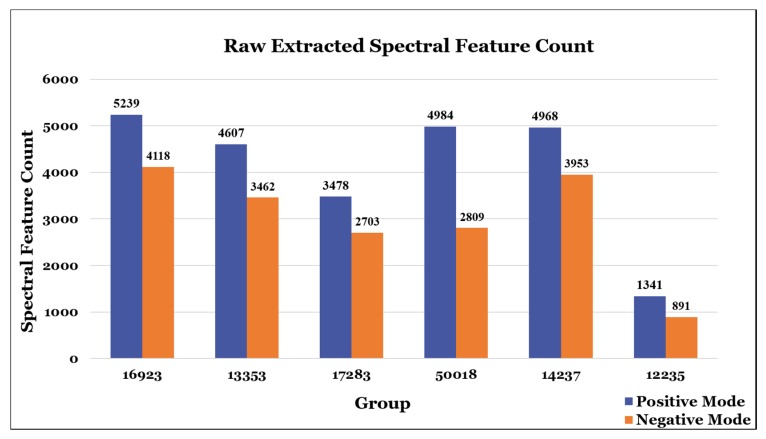
Raw extracted spectral feature count illustrating the total number of extracted positive and negative mode features found in each of the 6 participants that provided raw data. There are significant disparities in these statistics, with some participants extracting more than 4 times the number of features than others. While it is apparent that more positive mode features were extracted than negative, which adheres to what is typically observed in metabolomics data, this trend makes the results in Figure 4 even more striking, as the degree of overlap between positive mode features is far lower than that of negative mode features.

With such a paucity of overlap in the results, analysis of the raw extracted peak lists was also conducted. An identical agglomerative spectral feature clustering analysis was conducted on six of the original eight participants who provided their raw peak lists. Though there were far more overlapping features found across all six lists (Figure 5), these results were more difficult to interpret as the disparities in raw extracted feature counts for each list were substantial (Figure 6). Nonetheless, it is clear that spectral feature concurrence and consensus were at a far lower rate than one would hope for, especially when considering that there were no wet lab or instrumentation-related reproducibility issues to account for.

## 4. MRG 2016 Study: Lessons Learned and Future Directions

Global metabolomic and lipidomic profiling offer promise as discovery tools for precision medicine approaches. Because this field is still emerging, there is an urgent need for standardization at every step of the workflow to allow for robust, reliable, and reproducible data interpretations. The goal of the MRG 2016 study was to determine the impact of data pre- and postprocessing methodologies on study outcome.

We chose a very simplistic study design where mouse urine metabolomic profiles were generated using an ultra-performance liquid chromatographic system online with a quadrupole time-of-flight mass spectrometer. For analysis of untargeted data, the raw data files are typically converted to a universal file format that can then be processed by a peak detection software like XCMS (Scripps, La Jolla, CA, USA) [8] or MZmine [9]. The extracted features are used for difference detection using a combination of data filtering and normalization techniques followed by statistical analyses [10]. The most significant features are subject to accurate mass-based database search for assignment of putative annotations.

Surprisingly, there was little overlap across laboratories with respect to the reported top 50 statistically significant urinary spectral features between the sham and 5 Gy irradiated mice. On the other hand, there was a reasonable overlap of the reported total number of raw extracted features among the participant laboratories. Because we had asked the participants to use their preferred data analysis methodologies routinely used in their laboratories, the differences in their results suggest that the software/ peak detection algorithm can generate varied results for the same data set. Spectral alignment methods are broadly divided into warping and segmentation methods. In principal, spectral data could be aligned before peak detection or spectral peaks could be aligned across samples once they have been detected using their *m*/*z* and/or retention time coordinates for LC-MS data [11]. This could be attributed, at least in part, to the parameter settings for peak detection, alignment, and retention time corrections that are typically set as per the instrument setting for the respective laboratory. On the other hand, default software parameter settings may also cause variability in peak detection and suboptimal results.

Variability can also result from data normalization approaches that are meant to remove unwanted systemic bias and deconvolution methodologies which involve defining the baseline and fitting peak shape that can introduce variability in the resultant intensity (area under the curve) for each feature. One of the constraints of the study was the inability to perform a direct comparison of the peak detection parameters and their influence on peak and spectral information. This was attributed to the variability across the pool of participants and small sample size. Martin et al. have reported on interplatform data acquisition for untargeted metabolomics study as part of the metabo-ring initiative [12]. They found differences in the number of spectral features after postprocessing of untargeted data between and within LC-MS and NMR platforms. However, they also found a convergence with respect to spectral information.

To our knowledge, the MRG 2016 study is the first attempt to tease out likely factors that contribute to downstream variability in the analysis of untargeted metabolomics data. More recently, some groups have reported strategies to avoid such variability that can potentially confound differential abundance analyses [10,13,14]. Standardization of the field is imperative for broad-based implementation of untargeted metabolomic approaches that have become a potent discovery tool for biomedical research.

## Supplementary Materials

The following are available online at https://www.mdpi.com/2218-1989/10/4/128/s1, Figure S1: SOP for sample preparation for UPLC-QTOF-MS analysis, Figure S2: Details of chromatographic method used for resolving urine samples, Figure S3: Mass accuracy determination at the start and end of the batch acquisition for ESI positive and negative modes, respectively, Figure S4: Blank runs to monitor sample carryover and baseline drifts across the batch, Figure S5: TIC overlay of pooled quality control samples for ESI negative mode data.
