# Supplementary material for: The ABRF Metabolomics Research Group 2016 Exploratory Study: Investigation of Data Analysis Methods for Untargeted Metabolomics
Source: Metabolites. 2020 Mar 27;10(4):128. doi: 10.3390/metabo10040128 (PMC7241086; doi:10.3390/metabo10040128)
Supplement: Supplementary file 1 [file metabolites-10-00128-s001.zip › Supplementary Material/MRG_Supplementary Figures_122319.pptx]

## Slide 1
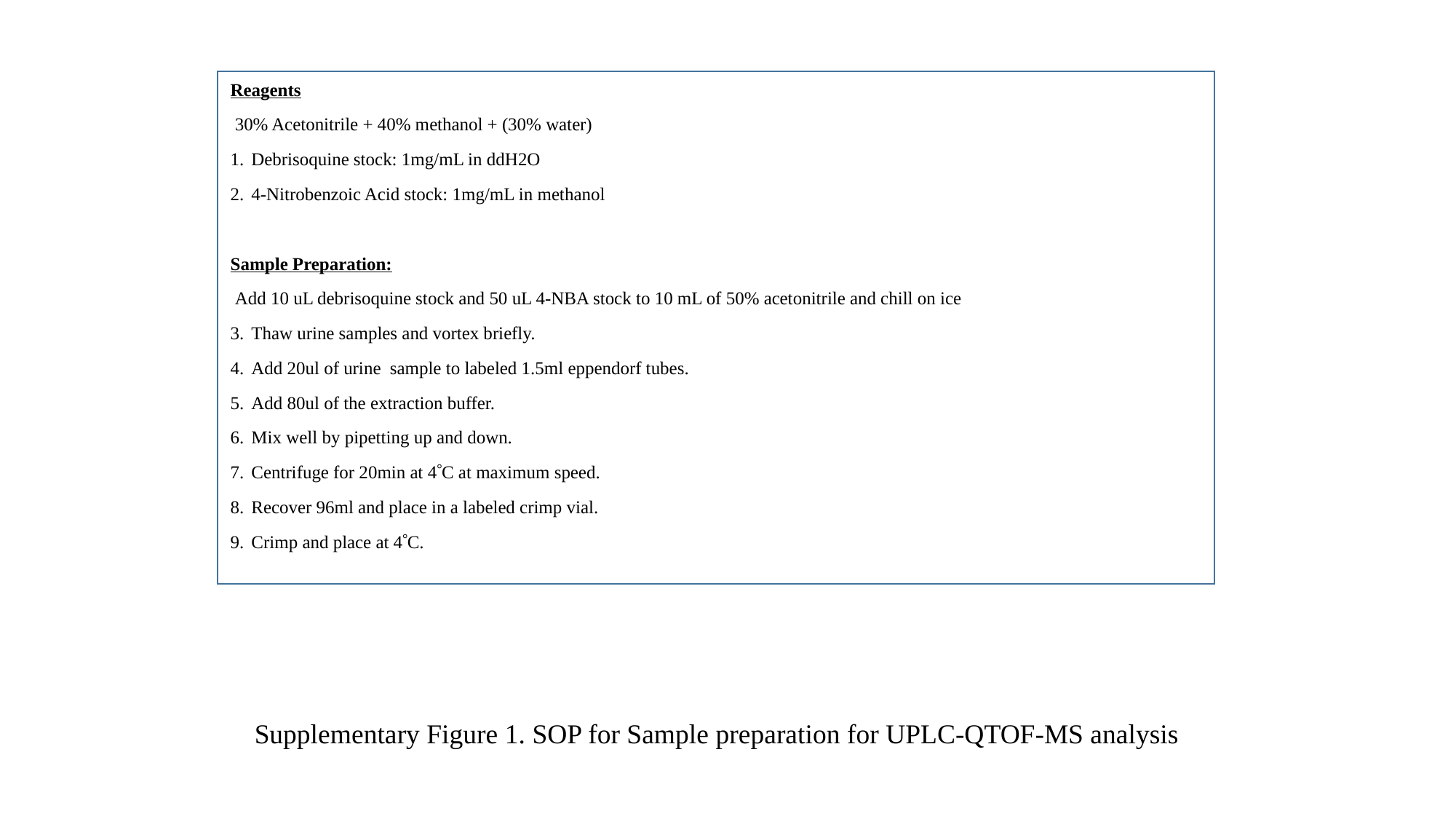

Reagents
 30% Acetonitrile + 40% methanol + (30% water)
Debrisoquine stock: 1mg/mL in ddH2O
4-Nitrobenzoic Acid stock: 1mg/mL in methanol
Sample Preparation:
 Add 10 uL debrisoquine stock and 50 uL 4-NBA stock to 10 mL of 50% acetonitrile and chill on ice
Thaw urine samples and vortex briefly.
Add 20ul of urine sample to labeled 1.5ml eppendorf tubes.
Add 80ul of the extraction buffer.
Mix well by pipetting up and down.
Centrifuge for 20min at 4°C at maximum speed.
Recover 96ml and place in a labeled crimp vial.
Crimp and place at 4°C.
Supplementary Figure 1. SOP for Sample preparation for UPLC-QTOF-MS analysis

## Slide 2
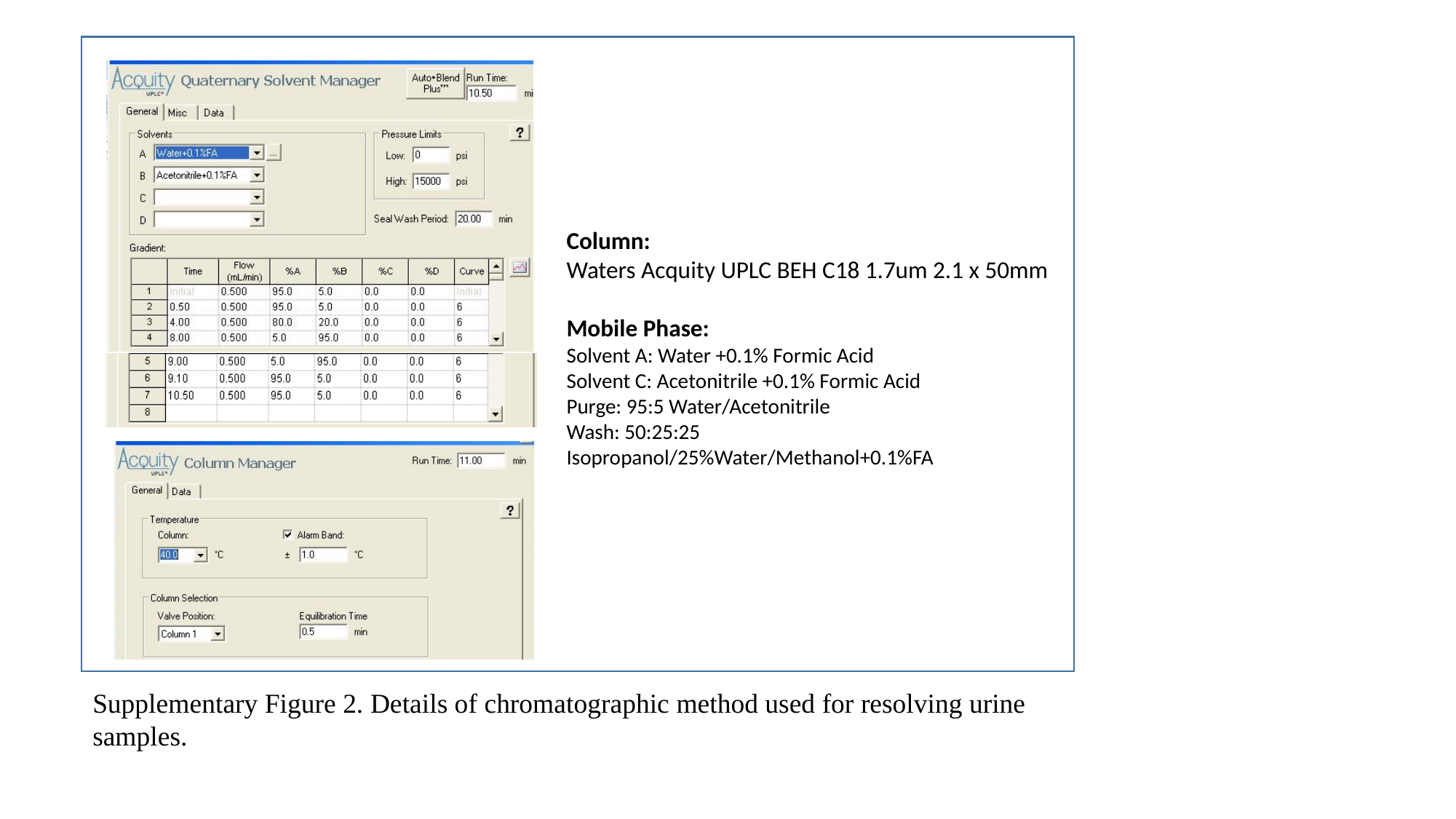

Column:
Waters Acquity UPLC BEH C18 1.7um 2.1 x 50mm
Mobile Phase:
Solvent A: Water +0.1% Formic Acid
Solvent C: Acetonitrile +0.1% Formic Acid
Purge: 95:5 Water/Acetonitrile
Wash: 50:25:25 Isopropanol/25%Water/Methanol+0.1%FA
Supplementary Figure 2. Details of chromatographic method used for resolving urine samples.

## Slide 3
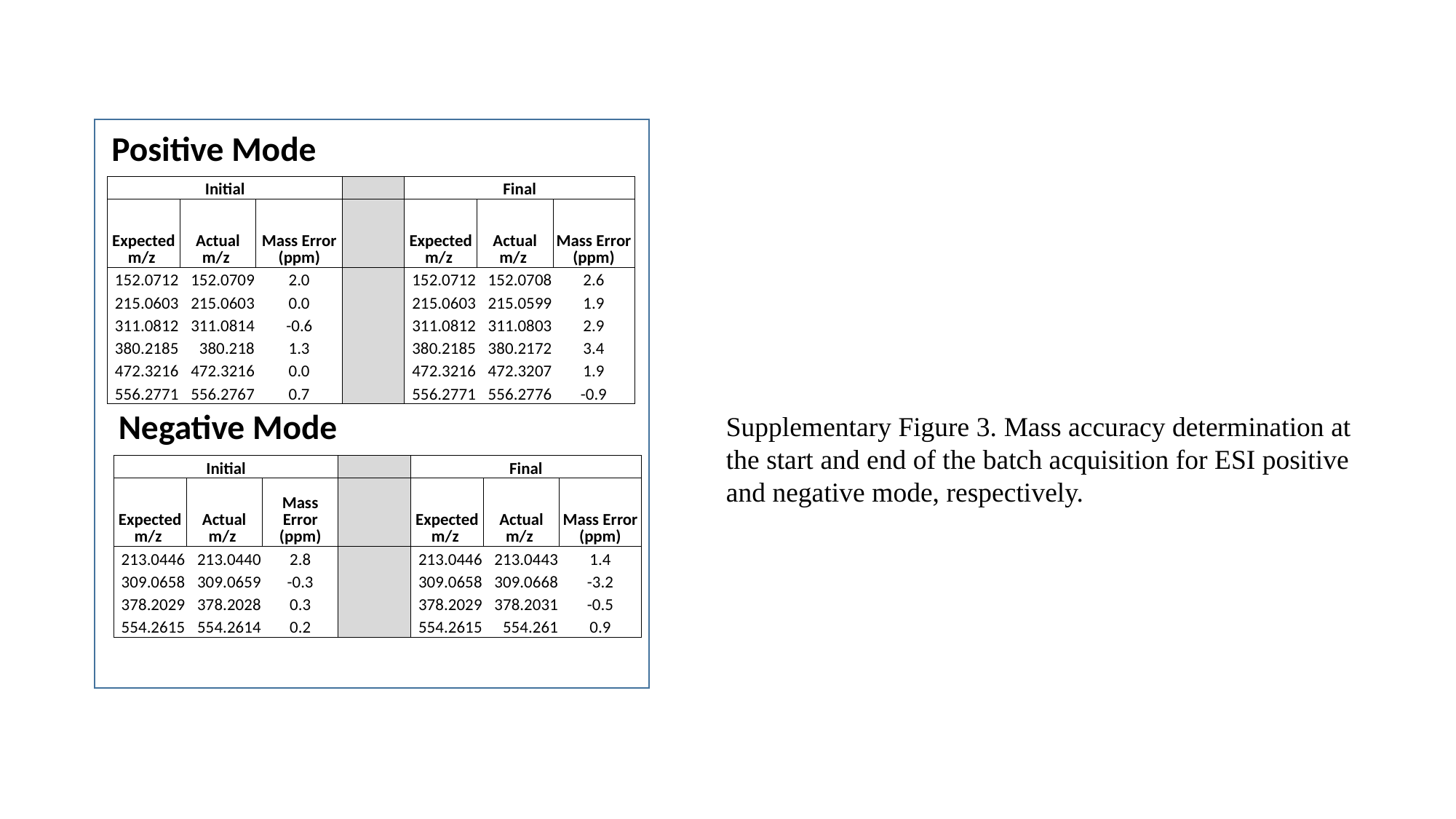

Positive Mode
| Initial | | | | Final | | |
| --- | --- | --- | --- | --- | --- | --- |
| Expected m/z | Actual m/z | Mass Error (ppm) | | Expected m/z | Actual m/z | Mass Error (ppm) |
| 152.0712 | 152.0709 | 2.0 | | 152.0712 | 152.0708 | 2.6 |
| 215.0603 | 215.0603 | 0.0 | | 215.0603 | 215.0599 | 1.9 |
| 311.0812 | 311.0814 | -0.6 | | 311.0812 | 311.0803 | 2.9 |
| 380.2185 | 380.218 | 1.3 | | 380.2185 | 380.2172 | 3.4 |
| 472.3216 | 472.3216 | 0.0 | | 472.3216 | 472.3207 | 1.9 |
| 556.2771 | 556.2767 | 0.7 | | 556.2771 | 556.2776 | -0.9 |
Supplementary Figure 3. Mass accuracy determination at the start and end of the batch acquisition for ESI positive and negative mode, respectively.
Negative Mode
| Initial | | | | Final | | |
| --- | --- | --- | --- | --- | --- | --- |
| Expected m/z | Actual m/z | Mass Error (ppm) | | Expected m/z | Actual m/z | Mass Error (ppm) |
| 213.0446 | 213.0440 | 2.8 | | 213.0446 | 213.0443 | 1.4 |
| 309.0658 | 309.0659 | -0.3 | | 309.0658 | 309.0668 | -3.2 |
| 378.2029 | 378.2028 | 0.3 | | 378.2029 | 378.2031 | -0.5 |
| 554.2615 | 554.2614 | 0.2 | | 554.2615 | 554.261 | 0.9 |

## Slide 4
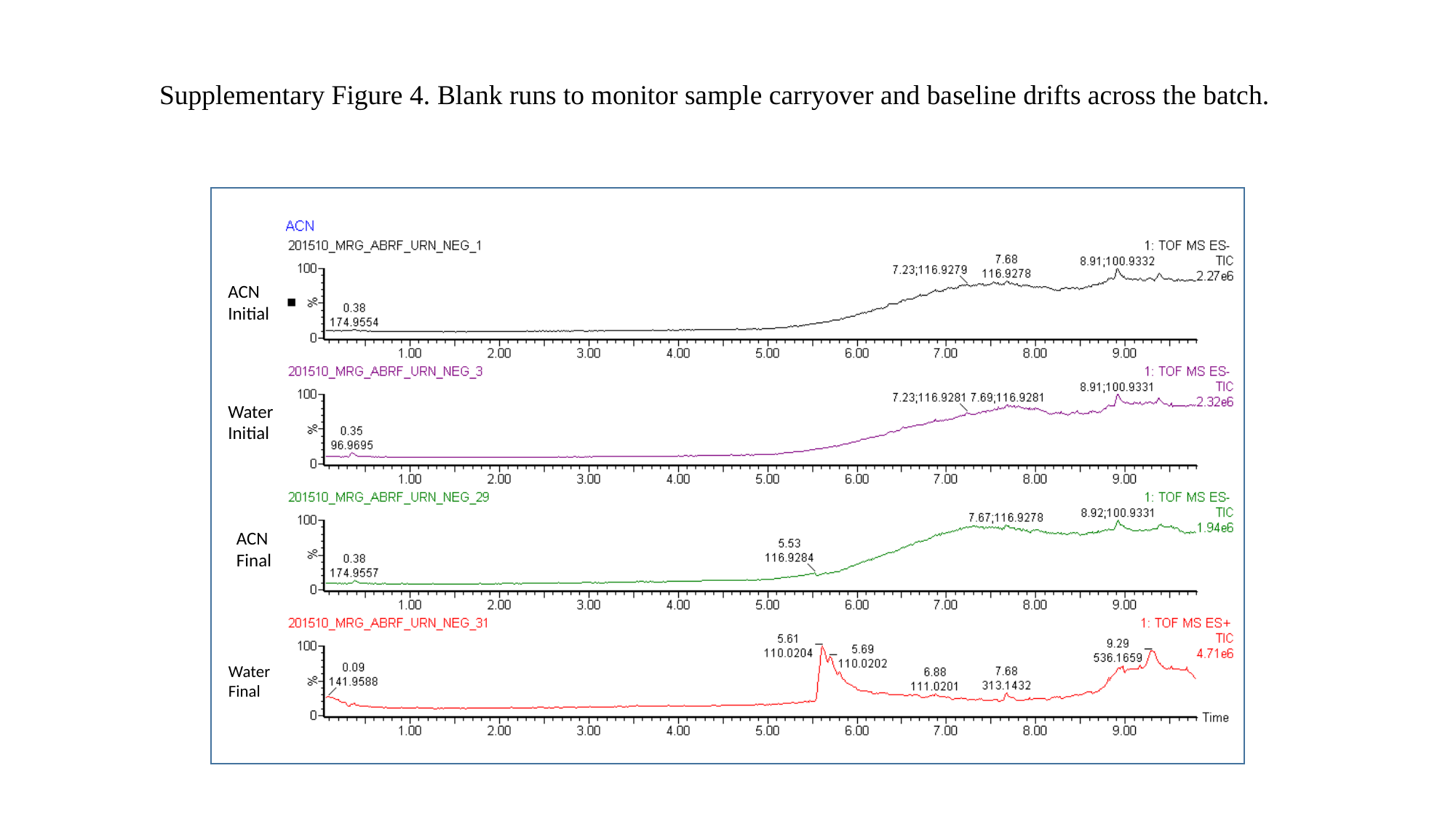

Supplementary Figure 4. Blank runs to monitor sample carryover and baseline drifts across the batch.
ACN Initial
Water Initial
ACN
Final
Water Final

## Slide 5
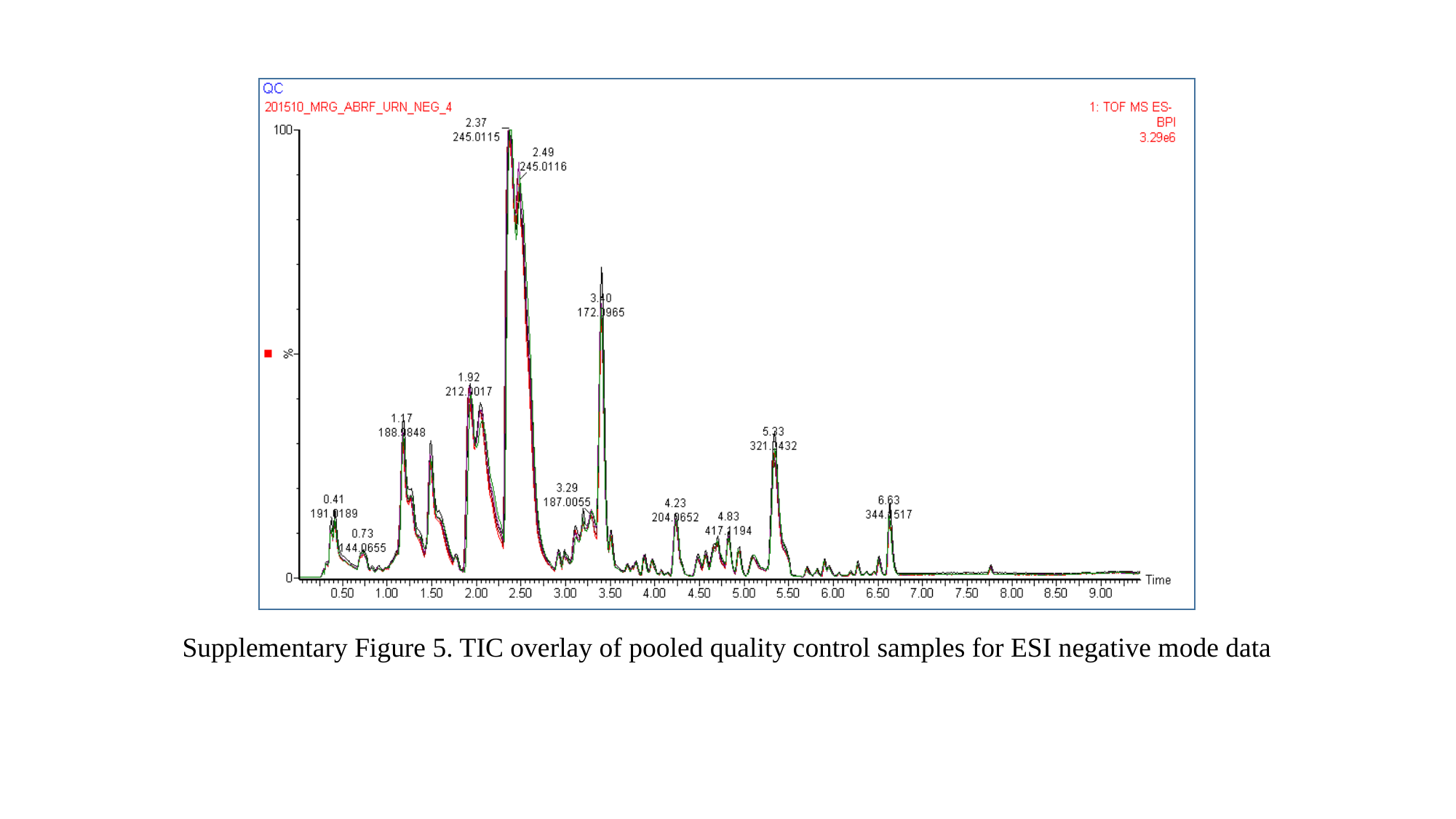

Supplementary Figure 5. TIC overlay of pooled quality control samples for ESI negative mode data
