# Supplementary material for: The ABRF Metabolomics Research Group 2016 Exploratory Study: Investigation of Data Analysis Methods for Untargeted Metabolomics
Source: Metabolites. 2020 Mar 27;10(4):128. doi: 10.3390/metabo10040128 (PMC7241086; doi:10.3390/metabo10040128)
Supplement: Supplementary file 1 [file metabolites-10-00128-s001.zip › Supplementary Material/Supp File 1_Pipeline_16923.pdf]

Data analysis ABRF_Met_2016: 16923

Generation of peak list:

Peak picking, Alignment:

- Progenesis QI, Version 2.2
- based on ".raw" data
- RT window 0.3-9.0min
- Possible adducts:
  - Positive: M+H, M+2H, M+3H, M+H-H2O, M+H-2H2O, M+Na, M+K, 2M+H, 2M+Na, 2M+K
  - Negative: M-H, M-2H, M-3H, M-H-H2O, M+FA-H, 2M-H, 2M+FA-H
- Normalization to given standards
- Results presented in "Peak detection" sheet

Data filtration before statistical analysis

Exclude features with the listed properties:

- RT < 0.5 min
- Peak width < 0.05 min
- Multiple charged ions
- CV > 30% in the samples of the QC group

The remaining 3663 (positive) or 3206 (negative) features were submitted to further statistical analysis.

Additionally, the accurate mass (5ppm error) and isotope pattern and adduct information was searched against the HMDB database (Version 3.6) for further identification. For 2423 (positive) or 2007 (negative) features at least one putative identification was assigned. Results are reported in the "Difference Detection" sheet.

Statistical analysis:

- Calculation of mean abundances of duplicate injection for each sample (inhouse R-script)

Multivariate analysis (SIMCA, Version 14, Umetrics AB)

- Data pretreatment:
  - Mean of duplicate injections
  - Non-transformed + Pareto scaled data or
  - log_10-transformed + Pareto scaled data
- OPLSDA with 10 groups for cross validation, 1 predictive + 1 orthogonal dimension:

- None of the 4 models (positive mode, negative mode, each with and without data transformation) separated the samples of the Irradiated (5Gy) and Sham (0Gy) group. Evaluation based on:
    - R^2, Q^2
    - permutation test
    - p-value of CV-ANOVA
- No discriminative features were found, no “Hit-List” created.

Univariate analysis (SIMCA, Version 14, Umetrics AB and inhouse Excel spread sheet):

- Data pretreatment:
    - Mean of duplicate injections
    - Non-transformed or log_10-transformed data
- Calculation of ANOVA p-values and Fold Changes (SIMCA)
- Calculation of False Discovery Rate according to Benjamini and Hochberg (1995)
- All resulting q-values > 0.05, except for 1 feature in positive mode: m/z 293.1008 at RT 5.31 min. However, further review of the peakshape of the feature revealed insufficient chromatographic properties.
- No discriminative features were found, no “Hit-List” created.

General comments

Raw data and peak picking:

- Reviewing the .raw data in MassLynx (Waters) indicated a series of saturated mass signals in the spectra, both in positive and negative ionization.
- The taken approach for peak picking (Progenesis QI) may not be optimal for centroid data. The applied filtration criteria were applied based on our profile-data experience. They may not be readily applicable to centroid data.
- 1 sample per group (0Gy_24H_3 and 5Gy_24H_14) exhibited generally lower abundances than the remaining samples of each group (TIC and BPI review). The reason could not be discovered. Hence, there was no rational (such as e.g. dilution error or other deviations in sample preparation) to exclude the samples from the analysis. The lower abundances were reproducible in the duplicate injections. Because the signal of the internal standard was well in the range of all samples, the normalization based on the internal standards did not compensate the observed lower overall TIC or BPI.

Experimental design:

- Comparing 5 and 5 samples per group, only, is challenging. The OPLS-DA is not the optimal approach for so small samples numbers and so many variables. It is especially challenging in cases of high intra-group variability as introduced by samples such as 0Gy_24H_3 and 5Gy_24H_14.

- The chosen sample sequence for analysis did not allow to reveal any systematic effect of a selected sample on its successor. Analyzing the 2nd replicates in a different order may have helped.
- The QC sample was analyzed 5 times within the sequence. Data evaluation without normalization to the internal standard indicated good reproducibility for the initial 3 injections, whereas the later injections indicated deviations (the last injection more than the second last QC injection). Due to the overall low number of injections, no conclusion for a systematic drift was drawn. At least 10 injections of the QC throughout the sample sequence can uncover drifts and allow for corrections using techniques such as LOESS. The intensity of the internal standard was relatively stable in the QCs. This may, however, not be representative for all features.

Statistical analysis:

- Lacking established own approaches for the analysis of duplicate injections, we involved the mean of the duplicate injections in our statistical analysis. Nested ANOVA, for instance, can consider technical replicates and could, thus, have made better use of the available information.
