# Supplementary material for: The ABRF Metabolomics Research Group 2016 Exploratory Study: Investigation of Data Analysis Methods for Untargeted Metabolomics
Source: Metabolites. 2020 Mar 27;10(4):128. doi: 10.3390/metabo10040128 (PMC7241086; doi:10.3390/metabo10040128)
Supplement: Supplementary file 1 [file metabolites-10-00128-s001.zip › Supplementary Material/Supp File 2_Pipeline_12235.pdf]

12235 – Overview of Data Processing

My background:

I have been involved in untargeted metabolomics for about 4 years. I have completed several large projects in a variety of species, although my expertise is in plants. I have used Waters instrumentation and am familiar with/ have the ability to read the .raw files provided but I chose (due to time restraints) not to start with the raw files or use the fragmentation data. I do not have experience working with urine, or really any mammal derived samples. Being unfamiliar with this type of sample and the expected metabolites, I was uncomfortable assigning IDs based solely on the exact mass especially since I did not see the spectrum to help determine the likely adduct. I did assign a few but I would not have reported them to anyone without followup analysis of the MSe data or targeted MS/MS. I also would have looked at the peaks to ensure that the peak was real and that the difference between the samples could be observed.

My process:

I started from the provided .cdf files that had been processed with XCMS. I calculated the p-value and fold-change using excel equations. I filtered to remove features with p-value > 0.05 and again to remove features with a fold change (in either direction) of <2.0. From the remaining features I combined the negative and positive features, sorted by fold change, removed obvious +1 and +2 isotopes and used the top 50 as my features of interest.

I used both Metlin and HMDB as the reference database. For adducts I allowed +/-H, Na, K, and NH4. I used the provided QC data as well as expected compounds such as creatinine to determine the mass difference tolerance (5 ppm).

I do not normally calculate a false discovery rate since we use p value and fold change, thus I said yes to all of the 50 features that I listed.
