# Supplementary material for: The ABRF Metabolomics Research Group 2016 Exploratory Study: Investigation of Data Analysis Methods for Untargeted Metabolomics
Source: Metabolites. 2020 Mar 27;10(4):128. doi: 10.3390/metabo10040128 (PMC7241086; doi:10.3390/metabo10040128)
Supplement: Supplementary file 1 [file metabolites-10-00128-s001.zip › Supplementary Material/Supp File 3_Pipeline_14237.pdf]

MRG2016 Data Analysis Summary (14237)

The data analysis was performed using custom prototype software and manual curation. The data processing started with the original raw files which were converted to mzXML format using the msConvert tool (ProteoWizard). Every 17th scan starting with the first scan in the converted data files was removed as they appeared to be unrelated to the sample (every 46th scan starting with the first scan was removed from the converted MS^E data files). Scans were numbered consecutively starting from 1 after the removal of the apparently unrelated scans. Peak integration bounds are reported using these indices. Peak finding was not exhaustive but was rather aimed at chromatographic peaks with potential significant differences between the two groups of replicates. These non-exhaustive peak lists are submitted as supplementary tables and contain 4968 peaks from positive mode and 3953 peaks from negative mode. Chromatographically unresolved peaks are reported as a single peak unless one of the unresolved peaks shows a different profile of peak areas across the samples compared to the other unresolved peak(s). The technical replicate samples were used thus making the analysis a comparison of two groups of ten replicates. Detected chromatographic peaks of putative related ions originating from the same compound (isotopologues, adducts, multimers, in-source fragments) were grouped based on similarities of their chromatographic profiles within single data files, similarities in the their relative peak areas across different samples, and possible chemical relationships. These pure component spectra were matched between positive and negative polarity modes based on similarities in retention times and possible chemical relationships. Peak integration was performed using a simple trapezoidal method between integration bounds without any chromatographic smoothing or baseline subtraction. Peak integration window of ±30 ppm was used. Peak areas were normalized to peak areas of the corresponding internal standards. Normalized peak areas of "characteristic" ions (mostly [M+H]+ or [M-H]-) were used for comparisons even if normalized peak areas of other corresponding ions (e.g. isotopologues) showed a more significant difference. T-test, although probably not the most appropriate test in this context, was used as a rough approximation of statistical significance of differences between peak areas of "characteristic" ions. It is not clear to the author, who is not a statistician, how to properly model the FDR for this type of data therefore the corresponding field is left blank. Putative chemical formulae were assigned to putative compounds based on m/z values of their ions and relative abundances of isotopologues. Putative identities were assigned based on MS/MS spectra (where available) or characteristic in-source fragments from MS1 data. A pdf visualization of chromatograms, MS1 and MS/MS spectra along with putative identifications and notes for the most significant differences is also submitted as supplementary material.
