# Supplementary material for: The ABRF Metabolomics Research Group 2016 Exploratory Study: Investigation of Data Analysis Methods for Untargeted Metabolomics
Source: Metabolites. 2020 Mar 27;10(4):128. doi: 10.3390/metabo10040128 (PMC7241086; doi:10.3390/metabo10040128)
Supplement: Supplementary file 1 [file metabolites-10-00128-s001.zip › Supplementary Material/Supp File 4_Pipeline_56483.pdf]

201510_MRG_ABRF_URN_POS_2301.CDF appears to be corrupted: the xcms script (in R) indicated this by returning errors only when this file was included in the sample. The data from “20151010_MRG_ABRF_study_TOFMS xv.xlsx” was used to break the data into sample groups: “0G” and “5G”. Positive and negative polarity tests were run on each set of samples to yield four sets of data from the netCDF files: “0GNEG”, “0GPOS”, “5GNEG”, and “5GPOS”. A set of “QCPOS” and “QCNEG” values was also generated.

SAMPLE XCMS.R SCRIPT

```
##XCMS script, requires >v1.25.4
source("http://bioconductor.org/biocLite.R")
update.packages()

biocLite("xcms")
biocLite("pcaMethods")

rm(list=ls(all=TRUE))

library(xcms)
## change the following file path to the folder where you have all those CDF files saved.
CDFpath="C:/Users/syu/Desktop/MRG Data/MRG/CDF/0G/NEG"
setwd="C:/Users/syu/Desktop/MRG Data/MRG/CDF/0G/NEG"

list.files("CDF",path=CDFpath,recursive=TRUE, full.names=TRUE)
CDFfiles<-list.files("CDF",path=CDFpath,recursive=TRUE, full.names=TRUE)

#see type "?findPeaks.centWave" in R for settings for centWave
xsetpos<-xcmsSet(CDFfiles, method='centWave', ppm=10, peakwidth=c(60,180), snthresh=6,
prefilter=c(3,1000), integrate=1, mzdiff=.005, noise=20,
verbose.columns=TRUE,fitgauss=TRUE,mzCenterFun="wMean",polarity="negative",
nSlaves=4)
#?group.density for help
xsetpos<-group(xsetpos, bw=5, mzwid=0.015, minfrac=.5, minsamp=2, max=1000)
xsetpos
#xsetpos2<-retcor(xsetpos, method="obiwarp", profStep=1, plottype="deviation")
xsetpos2<-retcor(xsetpos, method="obiwarp", profStep=1)
#xsetpos2<-retcor(xsetpos, plottype="deviation")
xsetpos2
xsetpos2<-group(xsetpos2, bw=5, mzwid=0.0015, minfrac=.5, minsamp=2, max=1000)
xsetpos3<-fillPeaks(xsetpos2)
xsetpos3
ptpos <- peakTable(xsetpos3)
#see ?write.table for help
write.csv(ptpos, "C:/Users/syu/Desktop/MRG Data/MRG/CDF/0G/NEG/MRG_0G_NEG.csv")
```

To determine possible adducts, the m/z values generated were fed into the METLIN database. The following methodology was used on each of the four groups of results to determine possible identities and adducts:

- For positive polarity:
  - M+H and M+Na were possible adducts
  - M+H was preferred over M+Na
  - The lowest METLIN ID# was used out of the preferred adduct to identify the metabolite
- For negative polarity:
  - M-H and M+Fa-H were possible adducts
  - M-H was preferred over M+Fa-H
  - The lowest METLIN ID# was used out of the preferred adduct to identify the metabolite

The following script was then used for positive samples and then negative samples in two runs to obtain t-test p-values. The result was exported to a text file. A two sample unpaired t-test with bonferroni correction was applied to generate the p-values. FDR was also calculated here.

SAMPLE TTEST.R SCRIPT

```
# source(..., print.eval=TRUE) to show all output, echo=TRUE to show commands

rm(list=ls())

setwd("/Users/syu/Desktop/04192016/CSV")
getwd()

# grab mz column data from csv's
df0 <- read.csv("0GNEG.csv")
df5 <- read.csv("5GNEG.csv")

sink(file = "NEG.txt")

cat("Negative Mode: MRG Individual Data", "\n")
cat("No Correction","\n")
ncol <- ncol(df0)

for (i in 1:nrow(df0)) {
  assign("x", df0[i,c(10:ncol)])
  assign("y", df5[i,c(10:ncol)])
  cat(t.test(x,y)$p.value, "\n")
}

cat("\n", "Bonferroni", "\n")

for (i in 1:nrow(df0)) {
  assign("x", df0[i,c(10:ncol)])
```

```
  assign("y", df5[i,c(10:ncol)])
  z <- t.test(x,y)$p.value
  assign(paste("p",i,sep=""), p.adjust(z, method = "bonferroni"))
  cat(get(paste("p",i,sep="")),"\n")
}

cat("\n", "FDR", "\n")
for (i in 1:nrow(df0)) {
  assign("x", df0[i,c(10:ncol)])
  assign("y", df5[i,c(10:ncol)])
  z <- t.test(x,y)$p.value
  assign(paste("p",i,sep=""), p.adjust(z, method = "fdr"))
  cat(get(paste("p",i,sep="")), "\n")
}

cat("\n", "\n", "No Correction", "||", "bonferroni", "||", "FDR", "\n")
for (i in 1:nrow(df0)) {
  assign("x", df0[i,c(10:ncol)])
  assign("y", df5[i,c(10:ncol)])
  cat("||", i, ":")
  cat(t.test(x,y)$p.value)
  z <- t.test(x,y)$p.value
  assign(paste("p",i,sep=""), p.adjust(z, method = "bonferroni"))
  cat("||", get(paste("p",i,sep="")))
  assign(paste("p",i,sep=""), p.adjust(z, method = "fdr"))
  cat("||", get(paste("p",i,sep="")), "||", "\n")
}

sink(file = NULL)
```

The results were pasted into the results (xlsx) file. If the m/z value was not present in both the “0G” and “5G” samples it was not included in the final analysis.
