# Supplementary material for: The ABRF Metabolomics Research Group 2016 Exploratory Study: Investigation of Data Analysis Methods for Untargeted Metabolomics
Source: Metabolites. 2020 Mar 27;10(4):128. doi: 10.3390/metabo10040128 (PMC7241086; doi:10.3390/metabo10040128)
Supplement: Supplementary file 1 [file metabolites-10-00128-s001.zip › Supplementary Material/Supp File 5_Pipeline_22014.pdf]

5-digit code: 22014

1. Peak Detection

Peak detection was attempted by using XCMS locally installed on a Linux server. The following parameters were chosen derived from the XCMSonline method for UPLC+Water QTof MS:

```
xset1 <- xcmsSet(method="centWave", peakwidth=c(2, 25), ppm=15, prefilter=c(3,500),
mzdiff=0.01, snthresh=10, integrate=2, nSlaves=24) xset2 <- group(xset1,
mzwid=0.015, minfrac=0.5, bw=5)
xset3 <- retcor(xset2, method="obiwarp", profStep=1, plottype = "deviation")
xset4 <- group(xset3, mzwid=0.015, minfrac=0.5, bw=5)
xset5 <- fillPeaks(xset4, nSlaves=24)
```

Already in the first peak picking step an error message was returned:

```
"error in checkForRemoteErrors(val) :   one node produced an error: m/z
sort assumption violated ! (scan 897, p 19137, current 1010.4774
(I=238.63), last 1010.4932)"
```

Also XCMSonline returned an error message and could not process the netcdf-files. This problem could not be solved. The following analysis was thus carried out using the provided xcms output.

2. Statistical Analysis

The statistical analysis was carried out by using a combination of a R script and MetaboAnalyst.

Positive mode data were normalized using the feature 114.066386781816_0.447378868102323. Only this feature matched the exact mass within the error margin of 5ppm and the retention time was within the expected range. Usually a matching of the MS2 data would + comparison to an authentic standard measured on the same setup would have been necessary. For the negative mode data, no feature corresponding to creatinine could be identified, neg. mode data were therefore not normalized.
To remove technical or chemical noise from the data, the coefficient of variation was calculated for the 5 QCs (replicates) and the 10 samples (each with two replicates, from both sample groups). As a rule, features were removed when CV(QCs)>CV(samples). In this case the technical variability of the QC sample replicates that were measured at three positions (beginning, middle, end) of the sequence was higher than any potential biological variability, justifying removal of these features. Around 6% of the pos. mode features and 18% of the neg. mode features were removed.

A PCA analysis was carried out using row-wise normalization across samples for each mZRT variable to max=1 prior to PCA.
The PCA showed that the QC samples were centered as expected, nevertheless showing a certain drift following the sequence of measured samples. The first three QCs and the two later once (in the sequence) are separated in PC1, nevertheless the effect is not too dramatic, so no drift correction was used. The technical replicates cluster nicely together. Taken together these data suggest that the UPLC/MS part of the experiment went fine without creating much bias.
Looking at the two sample groups (+/- radiation) this cannot be said about the biological samples and the mouse experiment that was carried out. The two groups are not properly separated,

5Gy_24H_17 and 5GY_24H_18 are more similar to the 0Gy samples than to the other three 5Gy samples. It is not possible to define an outlier at this stage, because two of five biological replicates of the irradiated group seem to behave differently. So no sample was removed accepting that statistical power will be reduced.

To carry out a statistical test, the feature intensities were first log transformed to approximate a normal distribution. A standard t-test was carried out, p-value was corrected for multiple testing with the FDR criterion and fold change was calculated. Prerequisite for significance was FDR < 0.05 and FC >2. In total 28 features (taken together from pos. and neg. mode) could be identified as significant. Metabolite identification was attempted by using the exact masses and the batch metabolite search of the Metlin database with 5 ppm accuracy setting. Ten metabolites could be putatively identified.
