# Supplementary material for: The ABRF Metabolomics Research Group 2016 Exploratory Study: Investigation of Data Analysis Methods for Untargeted Metabolomics
Source: Metabolites. 2020 Mar 27;10(4):128. doi: 10.3390/metabo10040128 (PMC7241086; doi:10.3390/metabo10040128)
Supplement: Supplementary file 1 [file metabolites-10-00128-s001.zip › Supplementary Material/Supp File 6_Pipeline_17283.pdf]

Data:

The RAW data from the XCMS spreadsheets were feature wise median centered and logarithmised for the statistical analysis.

A T-test model was calculated using Profile.Analysis 2.1 from Bruker Daltonics.

To find the Top 50 changed metabolic features, the data was filtered according to the uncorrected p-value (p>=0.001; 1 out of 1000 is a false positive). Further the remaining features were sorted by their FDR value (Yes <=0.05; No>0.05) and the absolute ratio. From this list the first 50 were selected.

The absolute ratio was calculated using the data that was normalized to the respective internal standard.

m/z were looked up using excel in a database containing all entries from the HMDB early 2016 (only M+H or M-H).

For visualization RStudio (0.99. 902) with R (3.3.1) was used:

PCA was created using the package pcaMethods 1.64.0
