# Supplementary material for: The ABRF Metabolomics Research Group 2016 Exploratory Study: Investigation of Data Analysis Methods for Untargeted Metabolomics
Source: Metabolites. 2020 Mar 27;10(4):128. doi: 10.3390/metabo10040128 (PMC7241086; doi:10.3390/metabo10040128)
Supplement: Supplementary file 1 [file metabolites-10-00128-s001.zip › Supplementary Material/Supp File 7_Pipeline_13353.pdf]

Materials and Methods

Peak picking was performed using available CDF files and xcms 1.48.0 (R 3.3.0). In short, CDFs were imported and processed using xcms function xcmsSet() with parameters set as follows: method="centWave", ppm=25, peakwidth=c(5,15), prefilter=c(5,500), noise=100). Detected peaks were filtered using a custom R function based on the zigzag index (Zhang and Zhao 2014), keeping only peaks with a zigzag value < 0.5. Retention times were corrected using group() followed by retcor() using method "loess". Final peak grouping across chromatograms was performed using group() with parameters bw=1, minfrac=0.25 and mzwid=0.02, and missing peaks were filled with fillPeaks(). Files from positive and negative mode were analyzed separately. Blank analyses were omitted.

Deconvolution, i.e. combination of ions belonging to the same compound, was performed with CAMERA (Kuhl et al. 2012) version 1.28.0, using functions xsAnnotate(), groupFWHM(), findIsotopes(), groupCorr() and findAdducts() with parameters set as follows:

xsAnnotate	sample=NA, polarity="positive" / polarity="negative"
groupFWHM	sigma=6, perfwhm=0.6, intval="maxo"
findIsotopes	maxcharge=2, maxiso=4, ppm=5, mzabs=0.01
groupCorr	cor_exp_th=0.75, graphMethod="lpc", calcIso=T, calcCiS=F, calcCaS=T
findAdducts	ppm=5, mzabs=0.01

Detected pseudospectra were filtered, discarding spectra with less than 3 ions. Remaining pseudospectra were submitted to annotation and statistical analysis as follows.

Mass hypotheses were generated using a custom R script that evaluates CAMERA adduct and isotope annotation and further spectral rules. These mass hypotheses were matched against a local copy of HMDB 3.6, for which in silico spectra were predicted with CFM-ID (Allen et al. 2014). Candidate structures were obtained within a mass error window of 0.01 Da and ranked by spectral similarity using xcms function specDist.meanMZmatch(). Matches with a similarity of 0 (no peak overlap) were discarded, all others were reported in decreasing order of similarity.

For the final peak table, intensity values were extracted for the base peak of each pseudospectrum, using the "into" value (peak area) of xcms.

For statistical analysis, negative and positive peak tables were combined. Intensity values were log10 transformed and normalized using an ANOVA model with terms "treatment", "median peak intensity", "internal standard" and "runorder" (TR+MP+IS+Order). Corrected values were obtained by eliminating the variances related to the latter three terms.

Differential metabolites were searched using 3 methods:

(1) Using a t-test per metabolite between 5 raw data mean values (of two technical replicates each) from 0G and 5G group, respectively
(2) Dto., for normalized data
(3) Using *p* values for "treatment" factor based on above ANOVA model (TR+MP+IS+Order) using raw data

In addition, a restricted PCA was calculated based on the normalized data, including only 83 metabolites passing a 0.01 significance threshold without correction for multiple testing.

Key findings

We report the main findings from above analysis.

Initial peak picking with xcms resulted in 4607 mass spectral features for ES-positive mode, and 3462 features for negative mode samples. These were combined (deconvoluted) to 2045 and 1423 pseudospectra, respectively, of which 354 and 330, respectively, passed the 3 ion filter and were kept for downstream analysis.

The median log-transformed peak area per sample was checked as a proxy for internal standard in negative/positive samples individually and combined. We conclude that

(1) samples from mouse 3 and 14 were apparently more diluted (lower average metabolite amount),
(2) technical reproducibility was high (low variation between replicates) and
(3) that positive mode internal standard (m/z 176.1187) was subject to strong variation (2-fold changes)

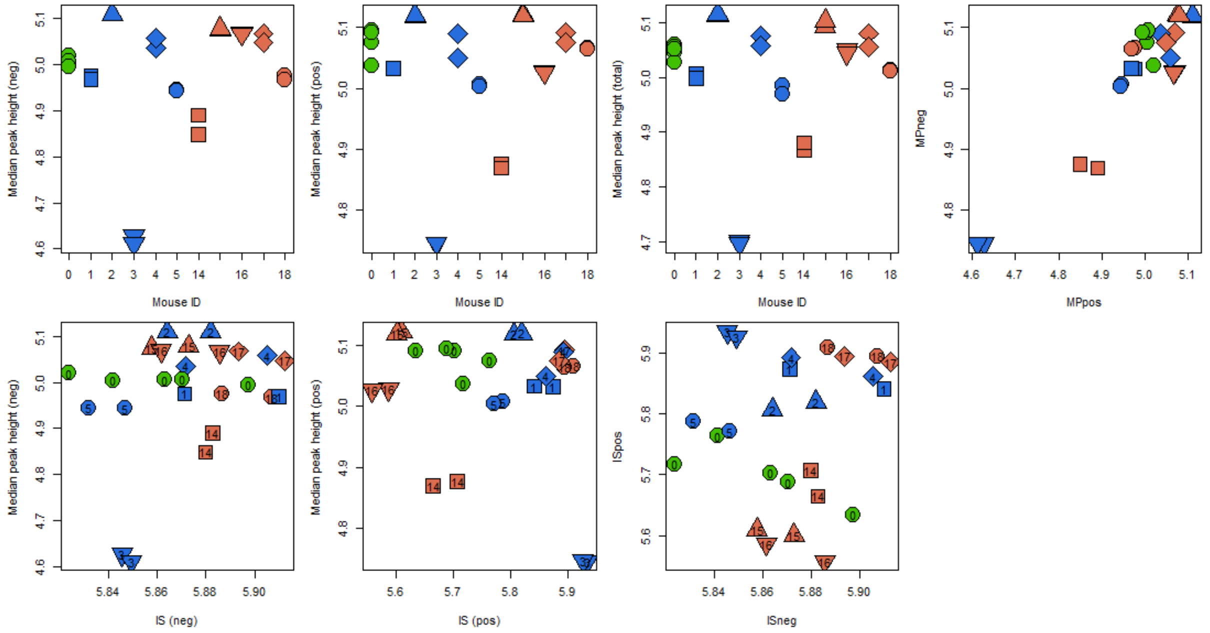

A PCA carried out on the raw (non-normalized) data confimed these observations and showed that Mouse 17 and 18 appeared more like the control group, rendering differential statistics difficult.

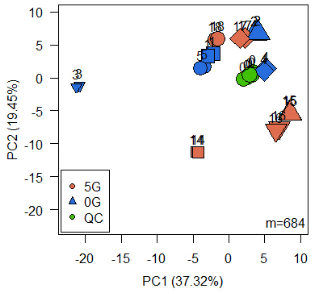

A PCA carried out on the normalized data resulted in better separation of the treated/non-treated groups:

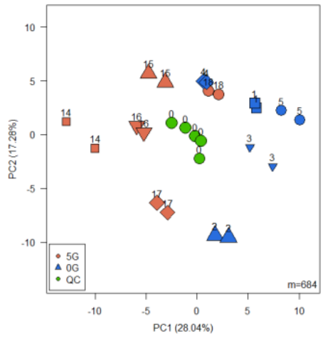

We noticed that about 30 peaks, e.g. “pos332”, were significantly different between QCs and remaining samples (which should not happen), but we kept them in the matrix as they do not result in false positives.

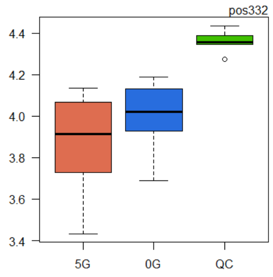

From the three approaches applied to find differentially regulated metabolites we conclude that

(1) ANOVA obviously has higher power as 20 instead of 10 mean values were used (resulting in smaller *p* values), and that
(2) FDR will be high in normalized data as effects are of moderate significance

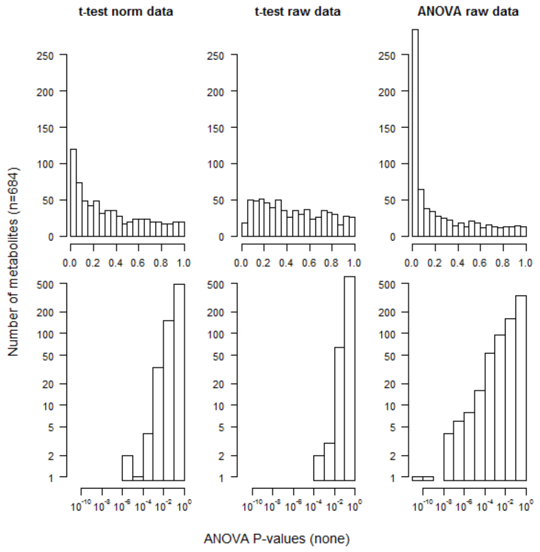

We show an example plot for the best differential candidate (showing box plot and individual data points of raw and normalized data on left and right respectively; *p* values for normalized data are

from t-test between replicate means and the ANOVA described above respectively).

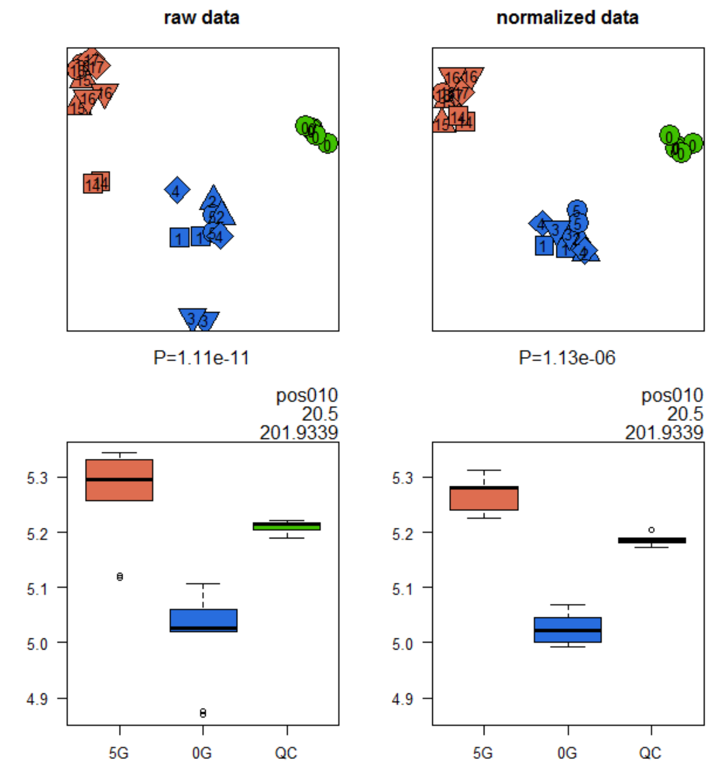

Finally, a restricted PCA, based on 83 significant metabolites only, reflected the experimental expectation, i.e. a significant effect of radiation treatment on urine metabolome:

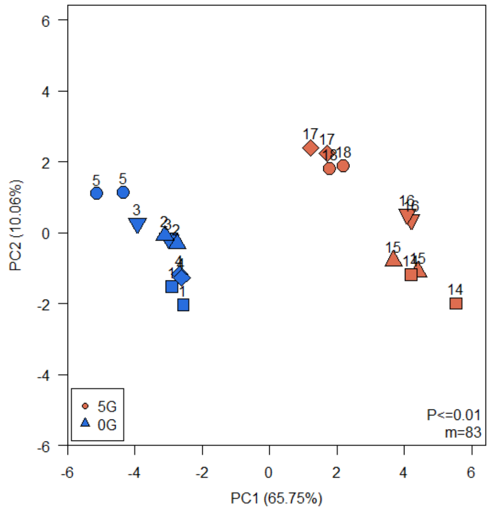

References

Allen, Felicity; Greiner, Russ; Wishart, David (2015): Competitive fragmentation modeling of ESI-MS/MS spectra for putative metabolite identification. In: *Metabolomics* 11 (1), S. 98–110. DOI: 10.1007/s11306-014-0676-4.

Kuhl, Carsten; Tautenhahn, Ralf; Böttcher, Christoph; Larson, Tony R.; Neumann, Steffen (2012): CAMERA. An Integrated Strategy for Compound Spectra Extraction and Annotation of Liquid Chromatography/Mass Spectrometry Data Sets. In: *Anal. Chem.* 84 (1), S. 283–289. DOI: 10.1021/ac202450g.

Zhang, Wenchao; Zhao, Patrick X. (2014): Quality evaluation of extracted ion chromatograms and chromatographic peaks in liquid chromatography/mass spectrometry-based metabolomics data. In: *BMC Bioinformatics* 15 (Suppl 11), S. S5. DOI: 10.1186/1471-2105-15-S11-S5.
