# Supplementary material for: The ABRF Metabolomics Research Group 2016 Exploratory Study: Investigation of Data Analysis Methods for Untargeted Metabolomics
Source: Metabolites. 2020 Mar 27;10(4):128. doi: 10.3390/metabo10040128 (PMC7241086; doi:10.3390/metabo10040128)
Supplement: Supplementary file 1 [file metabolites-10-00128-s001.zip › Supplementary Material/Supp File 8_Pipeline_50018.pdf]

Methods:

Raw data were submitted to XCMS online platform for analysis. A default method (UPLC-High Res POs (Waters)) was used. Feature detection setting is 15 ppm for maximal tolerated m/z deviation in consecutive scans, 2 s for minimum peak width, 25 s for maximum peak width. Both positive and negative data analysis used the same parameters except for polarity settings. Ion intensity (peak area) was extracted from the downloaded excel result and analyzed by the following steps.

1, Average value from two technical replicates of each sample was calculated. 5Gy (5 biological replicates) and 0Gy (5 biological replicates) were grouped based on the information MRG provided.

2, Data were imputed with normal distribution. P-value was calculated from Welch's unpaired t-test. Benjamini–Hochberg correction was used for adjusted p-value calculation.

3, Ratio was calculated using sum of the intensity values from each group.

4, MS1 match was done against the downloaded HMDB database using an in-house R script based on the accurate precursor mass.

Key findings:

1, 20 features have significant changes ($p <= 0.01$) in negative ion mode (14 up-regulated and 6 down-regulated in 5Gy group)

2, 42 features have significant changes ($p <= 0.01$) in positive ion mode (41 up-regulated and 1 down-regulated in 5Gy group)

3, Technical replicates are reproducible. But variations among biological replicates are big. Figure 1 shows the PCA graph for data from positive ion mode.

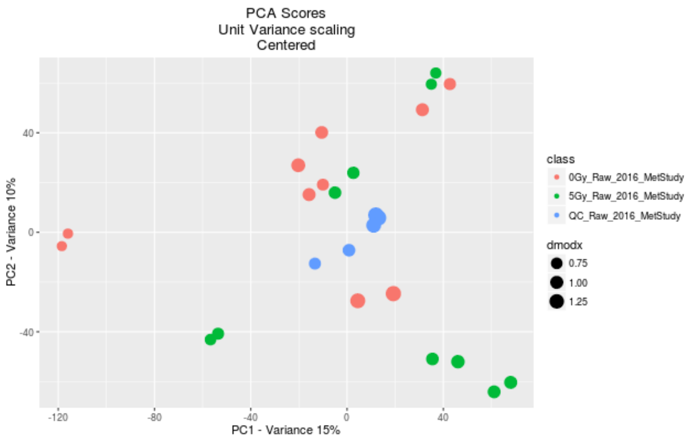

Figure 1: PCA graph for positive ion mode (technical replicates are included in the graph). Red dot: 0Gy; green dot: 5Gy; blue dot: quality control.
